# CTLA-2 Alpha Is a Potent Inhibitor of Angiogenesis in Murine Ocular Tissue

**DOI:** 10.3390/antiox10030456

**Published:** 2021-03-15

**Authors:** Kazuichi Maruyama, Kazuhito Yoneda, Sunao Sugita, Yoshimi Yamamoto, Masato Koike, Christoph Peters, Yasuo Uchiyama, Kohji Nishida

**Affiliations:** 1 Department of Vision Informatics, Osaka University Graduate School of Medicine, Suita 565-0871, Japan; 2 Integrated Frontier Research for Medical Science Division, Institute for Open and Transdisciplinary Research Initiatives (OTRI), Osaka University, Osaka 565-0871, Japan; knishida@ophthal.med.osaka-u.ac.jp; 3 Department of Ophthalmology, Kyoto Prefectural University Graduate School of Medicine, Kyoto 602-0841, Japan; kazuyone@koto.kpu-m.ac.jp; 4 RIKEN Center for Development Biology, Kobe 650-0047, Japan; sunao.sugita@riken.jp; 5 Laboratory of Biochemistry and Radiation Biology, Department of Veterinary Science, Faculty of Agriculture, Yamaguchi University, Yamaguchi 753-8511, Japan; yamataka@yamaguchi-u.ac.jp; 6 Department of Cell Biology and Neuroscience, Juntendo University Graduate School of Medicine, Tokyo 113-8421, Japan; mkoike@juntendo.ac.jp; 7 Institute of Molecular Medicine and Cell Research, University of Freiburg, 79098 Freiburg, Germany; christoph.peters@mol-med.uni-freiburg.de; 8 Department of Cellular and Molecular Neuropathology, Juntendo University Graduate School of Medicine, Tokyo 113-8421, Japan; y-uchi@juntendo.ac.jp; 9 Department of Ophthalmology, Osaka University Graduate School of Medicine, Suita 565-0871, Japan

**Keywords:** corneal inflammation, choroidal neovascularization, cytotoxic T lymphocyte antigen-2 alpha, angiogenesis

## Abstract

Cytotoxic T lymphocyte antigen-2 (CTLA-2) alpha has been reported to suppress the activities of cathepsin L (Cath L), which is deeply involved in angiogenesis. Therefore, we assessed whether CTLA-2 alpha plays a role in angiogenesis in ocular tissue. To establish models of corneal inflammation and experimental choroidal neovascularization (CNV), male C57BL/6J mice (*n* = 5) underwent corneal suture placement or laser-induced CNV, respectively. Mice were then injected with recombinant CTLA-2 alpha (1 µg) into the peritoneal cavity at day 0 and every 2 days after operation. In vitro experiments were performed to assess the inflammatory response by measuring TNF-alpha secretion in peritoneal cavity exudate cells (PECs) or the proliferation of mouse vascular endothelial cells (mVECs). CTLA-2 alpha treatment dramatically suppressed corneal angiogenesis, as well as laser-induced CNV. Moreover, CTLA-2 alpha inhibited the proliferation of mVECs in vitro, while CTLA-2 alpha abolishment was able to rescue proliferation. However, CTLA-2 alpha could not suppress cytokine secretion from inflammatory cells such as PECs. In summary, CTLA-2 alpha was able to suppress angiogenesis by suppressing endothelial cell proliferation. Further studies are needed to investigate its usefulness as a new antiangiogenic treatment for a variety of conditions, including age-related macular degeneration.

## 1. Introduction

Ocular tissues such as the cornea, retina, and especially the macular area, require an avascular condition to maintain good visual acuity. To protect against blindness, ectopic angiogenesis must be suppressed. Newly formed blood vessels are involved in many physiological processes and pathological conditions. For example, corneal neovascularization (CONV) and choroidal neovascularization (CNV), two forms of ocular angiogenesis, are major causes of blindness worldwide. CNV involves abnormal vessel growth from the choriocapillaris through the Bruch’s membrane, resulting in hemorrhage, scarring, exudation, and retinal detachment. The ultimate consequence of this neovascularization is severe loss of high-acuity central vision [1]. The molecular mechanisms underlying the development of CNV have not been fully elucidated, however the need for effective therapies to prevent and treat CNV is made even more urgent by the increase in the number of people aged over 65 years.

Importantly, vessel formation in the cornea is usually suppressed by several factors that maintain the clarity of the tissue [2,3], while several recent reports have demonstrated that endogenous angiogenic inhibitors are present in corneal epithelial cells. However, corneal hemangiogenesis—that is, abnormal vessel growth from the limbal (peripheral) vessel arcade—starts immediately following inflammation of the cornea [4]. The roles of macrophages in the induction of blood vessel formation at inflammation sites have been studied intensively, and some researchers have suggested that macrophages are primarily responsible for inducing blood vessel formation in response to inflammation. In particular, it is recognized that macrophages play important roles in CONV and CNV. CONV causes corneal opacification and consequent blindness, as well as the rejection reactions that sometimes follow corneal transplantation.

Antiangiogenic therapies use artificial antibodies or compounds extracted from plants or other natural sources [5]. Moreover, numerous angiogenic inhibitors that target vascular endothelial growth factor (VEGF) produced during CNV and CONV have been studied intensively [6]. CONV is mediated by members of the VEGF family, while VEGF induces CONV by binding to VEGF receptors (VEGFRs)-1 and -2 [6,7]. Specifically, VEGF-A binds to VEGFR-2, and this complex has emerged as the main inducer of CONV [6]. Previously, a molecular trap (VEGF TrapR1R2) was used to neutralize VEGF-A and block CONV [6]. Neutralization of VEGF-A not only inhibits CONV, but also interferes with the recruitment of inflammatory cells that produce many angiogenic cytokines in the cornea [6]. Thus, VEGF-A trapping has both direct and indirect antiangiogenic effects.

Notably, interactions between endothelial cells and the extracellular matrix (ECM), specifically components of the vascular basement membrane (VBM), play key roles in the regulation of angiogenesis [8]. Several ECM–VBM protein fragments with potent antiangiogenic properties have been isolated recently; these antiangiogenic properties are apparent only after proteolytic cleavage of the fragments from their respective parent molecules. These cryptic endogenous angiogenesis inhibitors specifically inhibit endothelial cell proliferation and cell migration in vitro and in vivo [9,10,11].

Cysteine proteinases involved in intracellular and extracellular protein degradation and turnover are found in a wide variety of organisms [12,13,14], as are protein inhibitors of cysteine proteinases [15]. Recently, a newly discovered class of cysteine proteinase inhibitors was reported [12,15]. Activated T cells and mast cells from mice express one such inhibitor, CTLA-2 alpha, which is highly similar to the pro-region of mouse cathepsin L (Cath L) [15]. Cath L comprises the catalytic classes of serine, aspartate, and cysteine peptidase that exhibit endo- or exopeptidase activity. There is growing evidence of specific intra- and extracellular functions of these lysosomal enzymes, which have been shown to critically influence tumor invasion and metastasis [16,17,18]. Moreover, Cath L is expressed in endothelial progenitor cells, which play a critical role in angiogenesis in vivo [19].

The purpose of the present study was to investigate whether CTLA-2 alpha could suppress angiogenesis in ocular tissue and to use recombinant CTLA-2 alpha to investigate the mechanisms mediating the suppression of angiogenesis. The results showed that CTLA-2 alpha downregulated vascular endothelial cell proliferation and that the use of an anti-CTLA-2 alpha blocking antibody was able to restore cell proliferation in vitro.

## 2. Materials and Methods

### 2.1. Animals

Male C57BL/6J mice (8 weeks old) purchased from Japan CREA (https://www.clea-japan.com/en.html, accessed on 12 February 2021, Shizuoka, Japan) were used for corneal suture placement and laser-induced CNV procedures, as described below. All animals were treated in accordance with the ARVO (The Association for Research in Vision and Ophthalmology) Statement on the Use of Animals in Ophthalmic and Vision Research, and all experiments were approved by the Committee for Animal Research of Kyoto Prefectural University of Medicine (M18-7).

### 2.2. Hybridization Probes

A full-length mouse cDNA sequence encoding CTLA-2 alpha was inserted into the pBluescript II SK plasmid. An approximately 350 base-pair (bp) fragment was amplified using polymerase chain reaction (PCR) and forward (5′-CATTCGGATCCGGCTGCTCCACCCCCTGATCC-3′) and reverse (5′- GCCAGGTACCTTACTCTGGCTAGCCCTTCC-3′) primers. This fragment was subcloned into a pGEM-T Easy vector (Promega Biosciences, Inc., San Luis Obispo, CA) containing promoters for T7 and SP6 polymerases. For in situ hybridization, sense and antisense digoxigenin (DIG)-labeled cRNA probes were generated using T7 and SP6 polymerases, respectively (DIG RNA Labelling Kit; Roche Diagnostics, Tokyo, Japan) (21).

### 2.3. Recombinant CTLA-2 Alpha

Recombinant CTLA-2 alpha was efficiently expressed in *E. coli* cells and purified using His-Bind affinity chromatography, as described previously [20]. The amino acid sequence of the recombinant CTLA-2 alpha was MGHHHHHHHHSSGHIEGRHMLEDPAAPPPDPSLDNEWKEWKTKFAKAYNLNEERHRRLVWEENKKKIEAHNADYEQGKTSFYMGLNQFSDLTPEEFKTNCYGNSLNRGEMAPDLPEYEDLGKNSYLTPGRAQPE.

### 2.4. Anti-CTLA-2 Alpha Antibody

Antisera against CTLA-2 alpha were obtained by immunizing rabbits against purified recombinant CTLA-2 alpha. Anti-CTLA-2 alpha IgG was affinity-purified using a recombinant CTLA-2-alpha bound to commercially available resin (HiTrap; Amersham Biosciences, Inc., Piscataway, NJ, USA).

### 2.5. Corneal Suture Placement

Incisions extending more than 120° from the corneal circumference were made in the stroma, then three 11–0 nylon sutures were inserted [21]. To obtain a standardized angiogenic response, the outer edge of each suture was placed halfway between the limbus and a line demarcated by a 2 mm trephine; the inner edge of each suture was equidistant from the 2 mm trephine line. The sutures were left in place for 7 days. The experimental group received a peritoneal injection of recombinant CTLA-2 alpha (1 µg; 200 µL) immediately and every 2 days after the operation. Seven days after the operation, the mice (each group, *n* = 5) were euthanized via cervical dislocation and the corneas were collected for staining. The experiment was performed twice.

### 2.6. Laser-Induced CNV

CNV was induced using a modified version of a previously described technique [22]. Briefly, mice were anesthetized with ketamine hydrochloride (100 mg/kg body weight) and the pupils were dilated with 1% tropicamide. Three burns from a 532 nm diode laser (50-mm spot size, 0.1 s duration, 120 mW) were delivered to each retina using the slit-lamp delivery system with the OcuLight GL Photocoagulator (Iridex Corporation, Mountain View, CA, USA) and a handheld cover slide as a contact lens. Burns were placed at the 6-, 9-, 12-, and 3-o’clock positions of the posterior pole of the retina. Production of a bubble at the time of laser delivery is an important factor in obtaining CNV [22], as it indicates rupture of the Bruch’s membrane; only burns that produced a bubble were included in the study. The experimental group received a peritoneal injection of recombinant CTLA-2 alpha (1 µg; 200 µL) immediately and every 2 days after the operation. Fourteen days after the operation, the mice (each group, *n* = 5) were euthanized with cervical dislocation and the eyeballs were collected for staining. The experiment was performed twice.

### 2.7. Determination of Hemangiogenesis

The corneas were excised, rinsed three times in PBS, and fixed in acetone for 1 h. They were then rinsed once again in PBS; blocked with 2% BSA–PBS; incubated overnight at 4 °C with rat antimouse CD31 (PECAM-1) (1:100; BD Biosciences Pharmingen, San Diego, CA, USA); then washed, blocked, and stained with FITC-labeled secondary antibody (1:100; Jackson ImmunoResearch Laboratories, Westgrove, PA, USA) for 1 h. Stained whole-mount sections were analyzed under an Olympus fluorescence microscope (Olympus Corporation, Tokyo, Japan) and a Leica TSC-SP2 inverted and upright confocal laser-scanning microscope (Leica Microsystems K.K., Tokyo, Japan). Digital pictures of the flat mounts were taken using a spot image analysis system, and the area covered by CD31-positive blood vessels on each section [23,24] was measured using NIH ImageJ software. The total corneal area covered by these vessels was outlined using the innermost vessel of the limbal arcade as the border. The area of blood neovascularization within the cornea was calculated and normalized to the total corneal area. These values were expressed as percentages of the cornea covered by vessels.

### 2.8. Determination of CNV

The sizes of the CNV lesions were measured in choroidal flat mounts [25] by an investigator who was blinded to the treatment group. Mice used for the flat-mount technique were anesthetized and perfused with 0.2 mL Concavalin-A (Con-A) and fixed with 25 mL of 4% phosphate-buffered formalin. The cornea and lens were removed and the entire retina was carefully dissected from each eyecup. Radial cuts (*n* = 4 to 7, average 5) were made from the edge to the equator, and the eyecup was flat-mounted in VECTASHIELD (Vector Laboratories, Inc., Burlingame, CA, USA) with the sclera facing down. Flat mounts were examined by fluorescence microscopy using an Olympus fluorescence microscope and a Leica TSC-SP2 inverted and upright confocal laser-scanning microscope. The CNV area was quantified with the CD31-positive staining area that outlined the fluorescent blood vessel. The digital image was captured and CNV identified by setting a threshold level of fluorescence (only the CD31-positive vessel was captured). NIH ImageJ was used to measure the total area of CNV associated with each area. Statistical comparisons were made between the sizes of lesions stained with CD31 in the experimental groups.

### 2.9. Mouse Pituitary Vasuclar Endothelail Cell Culture

Mouse pituitary vascular endothelial cells (mVECs) (RIKEN BioResource Center Cell Bank, Ibaraki, Japan) were cultured for 24 h at 37 °C in a 5% CO_2_ and air mixture at a density of 10^6^ cells/plate on 35 mm collagen-1-coated culture plates and in EGM-2 medium containing 10% bovine serum albumin (BSA) (Sigma-Aldrich, St. Louis, MO, USA), 1 × 10^−5^ M 2-mercaptoethanol (ME) (Sigma-Aldrich), 10 mM HEPES, 0.1 mM non-essential amino acid, 1 mM sodium pyruvate, 100 U/mL penicillin, and 100 μg/mL streptomycin (BioWhittaker, Inc., Walkersville, MD, USA). The cultured cells were then assayed by XTT (Cell Proliferation Kit, Roche, Mannheim, Germany) following stimulation with recombinant CTLA-2 alpha (in several concentrations).

### 2.10. Collection and Culture of Peritoneal Exudate Cells from the Peritoneal Cavity

Thioglycollate (4 mL; 3%) was injected into the peritoneal cavity of C57BL/6 mice. Peritoneal exudate cells (PECs) were collected from the peritoneal cavity 4 days after injection [26]. PECs were washed, re-suspended, and cultured (24 h at 37 °C in a 5% CO_2_ and air mixture) at a density of 10^6^ cells/plate on 35 mm culture plates in RPMI-1640 medium containing 10% BSA (Sigma-Aldrich), 1 × 10^−5^ M 2-ME (Sigma-Aldrich), 10 mM HEPES, 0.1 mM non-essential amino acid, 1 mM sodium pyruvate, 100 U/mL penicillin, and 100 μg/mL streptomycin (BioWhittaker). The culture supernatants were then assayed for tumor necrosis factor alpha (TNFα).

### 2.11. ELISA for TNF-Alpha

The concentration of TNF-alpha was assayed using a specific sandwich ELISA (Quantikine murine TNF-alpha; R&D Systems, Minneapolis, MN, USA). In brief, PECs were treated with lipopolysaccharide (LPS: LPS-EB Ultrapure from *E. coli* 0111:B4 strain-TLR-4 ligand, InvivoGen San Diego, CA, USA) (1 µg), CTLA-2 alpha (10 µg or 100 ng), or both. Culture medium samples of PECs and standard recombinant TNF-alpha were added to the wells of pre-coated 96-well plates and incubated for 2 h at room temperature. The plates were washed, TNF-alpha antibody conjugates were added to each well, plates were incubated for 2 h, and then washed again. Substrate solution was added to each well and plates were incubated for 30 min at room temperature. H_2_SO_4_ (1.0 N) was added to each well to stop the enzymatic reaction. A plate reader (MicroQuant; Bio-Tek Instruments, Winooski, VT, USA) was used to read the optical density at 450 nm so as to measure the colour change. The concentration of cytokines in each sample was calculated based on a standard curve generated using optical density measurements and the corresponding concentrations of a set of cytokine standards; all standards were run and measured in parallel with the samples.

### 2.12. Cell Proliferation Assay

XTT assays were used to measure cell viability; XTT reagent (Roche Diagnostics, Nutley, NJ, USA) was added for 6, 12, or 24 h at 37 °C, and the absorbance was read using an ELISA plate reader. The average absorbance measured for media plus treatment was subtracted from each test sample, based on a previous report [27]. Each experiment was performed at least three times on different days.

### 2.13. CTLA-2 Alpha and Cath L Overexpression in mVECs

To overexpress CTLA-2 alpha in mVECs, mouse CTLA-2-pIRES-EGFP and Cath L vectors were synthesized and the constructs were transfected into mVECs using transfection reagent (Effectene Transfection Reagent; Qiagen K.K., Tokyo, Japan). As a control, the pIRES vector alone was used for the assay. Fluorescence microscopy and quantitative reverse-transcription PCR (RT-PCR) were used to confirm that CTLA-2 alpha and Cath L were overexpressed.

### 2.14. Statistical Analysis

The Mann-Whitney test was used to assess the statistical significance of the differences in neovascularization (NV) scores and TNF-alpha measurements, and the Student’s *t*-test was used for differences in proliferation response. Here, *p*-values < 0.05 were considered significant.

## 3. Results

### 3.1. The Effects of CTLA-2 Alpha in the Corneal Suture Model

**Figure 1:**
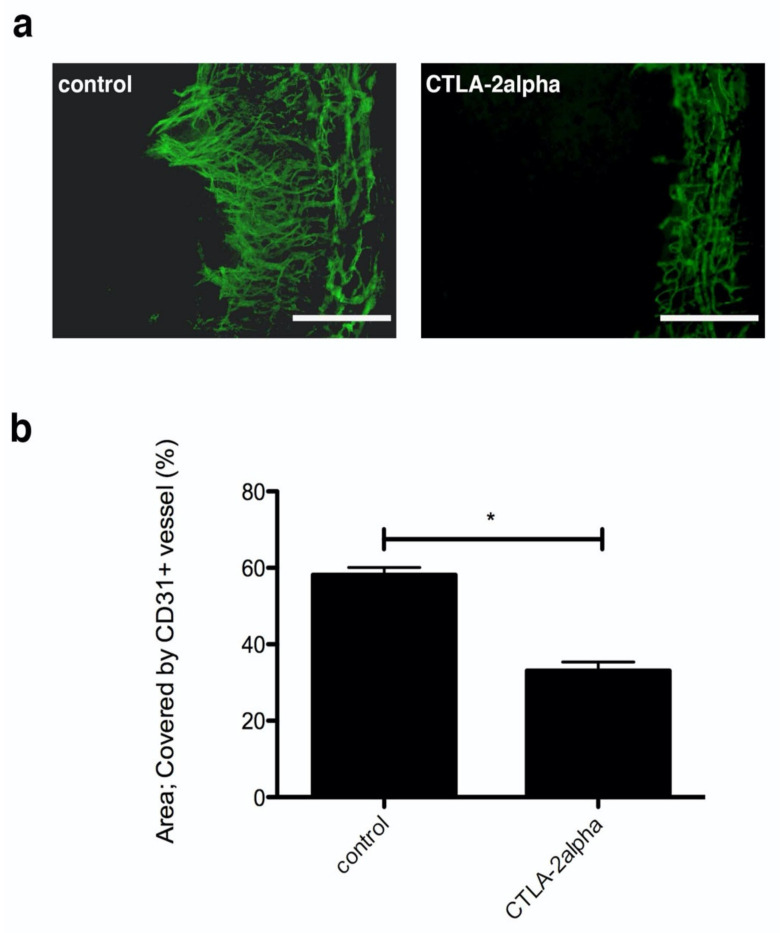
Corneal angiogenesis by suture placement in CTLA-2 alpha (Cytotoxic T-Lymphocyte-Associated protein 2) treatment. (**a**) Fluorescence microscopy image of CD31-positive staining (green), which represents corneal hemangiogenesis, 7 d after suture placement. (**b**) Corneal hemangiogenesis, measured by the area of CD31-positive staining, 7 days after suture placement. Scale bars, 75 µm. Note: * *p* = 0.0357.

The cornea usually inhibits blood vessel infiltration from the conjunctiva to maintain transparency. However, newly formed vessels will infiltrate the limbus and appear in the cornea after it has been wounded. Here, we placed sutures in the corneas to create wounds and induce blood vessel infiltration. After sutures were placed on corneas, newly formed vessels sprouted from limbal vessels, and the sprouting vessels reached the suture wound areas around day 7. This model of corneal wounding was used to investigate the effects of recombinant CTLA-2 alpha on hemangiogenesis in the cornea. Growth of CD31-positive blood vessels into the central cornea from the limbus in the PBS-treated mouse corneas was evident within 7 days of suture placement. Hemangiogenesis was quantified by measuring the area covered by newly formed vessels, specifically by measuring the area of CD31-positive staining (hemangiogenesis). Treatment with CTLA-2 alpha (1 µg/200 µL) significantly reduced the CD31-positive area (*p* = 0.0357; Figure 1). However, a higher concentration of CTLA-2 alpha did not affect suture-induced hemangiogenesis formation; specifically, the results from the PBS-treated group and the group treated with CTLA-2 alpha (10 µg/200 µL) were not significantly different.

### 3.2. The Effects of CTLA-2 Alpha in the CNV Model

**Figure 2:**
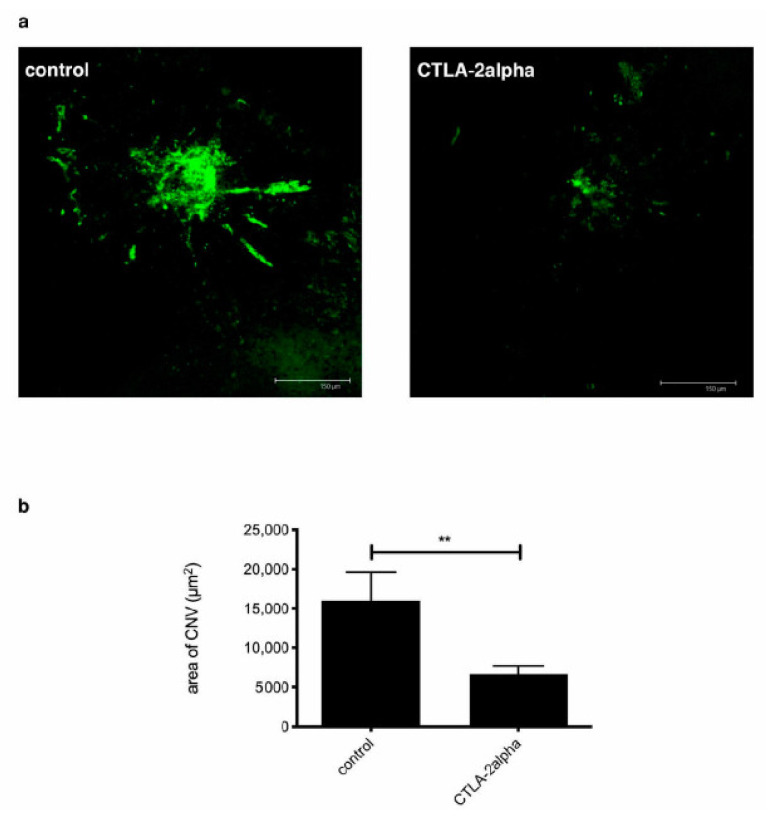
Laser-induced CNV (Choroid Neo Vasculalization) and the effects of CTLA-2 alpha treatment. (**a**) Confocal microscope image of tomato lectin staining, representing laser-induced CNV. (**b**) Laser-induced CNV, as measured by the area of tomato lectin staining 7 d after suture placement. Scale bars, 150 µm. ** *p* = 0.0100.

Laser-induced CNV was used as a model of age-related macular degeneration in order to verify whether recombinant CTLA-2 alpha could control this type of CNV. Within 7 days following laser treatment in the posterior of the eye, newly formed blood vessels had grown through the Bruch’s membrane and the retinal pigment epithelium (RPE) to the retina in PBS-treated mice, and the area of CNV-associated vessel growth was quantified by measuring CD31 staining. CNV was significantly suppressed by treatment with CTLA-2 alpha (1 µg/200 µL) compared with treatment with PBS (*p* = 0.0100; Figure 2a,b). However, we noticed that CNV, as with CONV, was not suppressed by a higher concentration of CTLA-2 alpha (≥10 µg/200 µL).

### 3.3. The Effects of CTLA-2 Alpha on PEC Activation In Vitro

**Figure 3:**
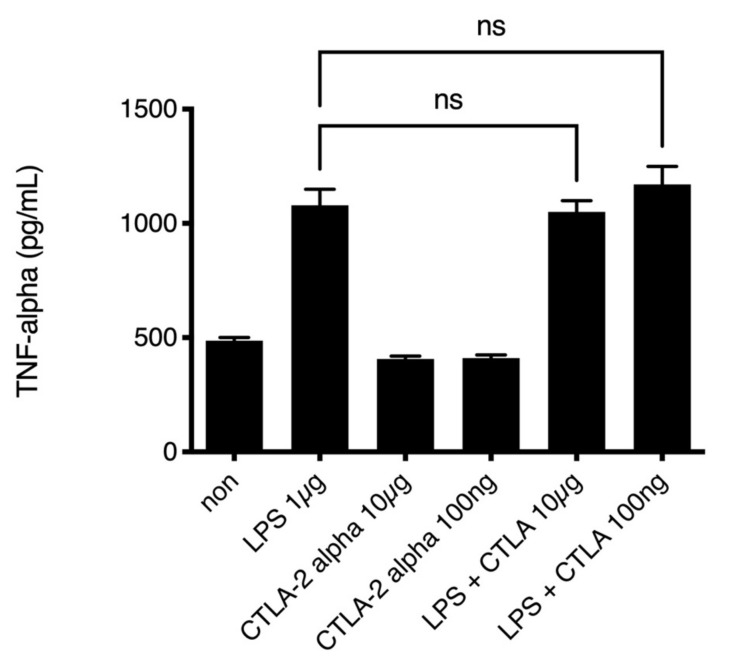
TNF-alpha secretion from peritoneal infiltrating cells. Peritoneal exudate CD11b and F4/80 double-positive cells were cultured with PBS (non as control), LPS (1 µg/mL), CTLA-2 alpha (10 µg/mL or 100 ng/mL), or both LPS and CTLA-2 alpha. TNF accumulation in the culture media was measured 24 h after stimulation using an ELISA kit. LPS: lipopolysaccharide; ns.: not significant difference.

Previously, we presented data indicating that reductive macrophages secrete high amounts of TNF-alpha [28]. We assessed whether CTLA-2 alpha suppressed TNF-alpha, a marker of inflammation, in PECs, given that PECs express F4/80 and CD11b on their surfaces [29] and the cells infiltrating the cornea also expressed both F4/80 CD11b. To assess the inflammatory response of PECs stimulated with LPS, we performed ELISA to measure TNF-alpha secretion from LPS-stimulated PECs. The levels of TNF-alpha in the low-dose and high-dose CTLA-2 alpha treatment groups were not different from those in the control group (Figure 3). This result showed that CTLA-2 alpha did not suppress TNF-alpha secretion, a marker of inflammation, in PECs at the site of inflammation.

### 3.4. The Effects of CTLA-2 Alpha on mVEC Proliferation In Vitro

**Figure 4:**
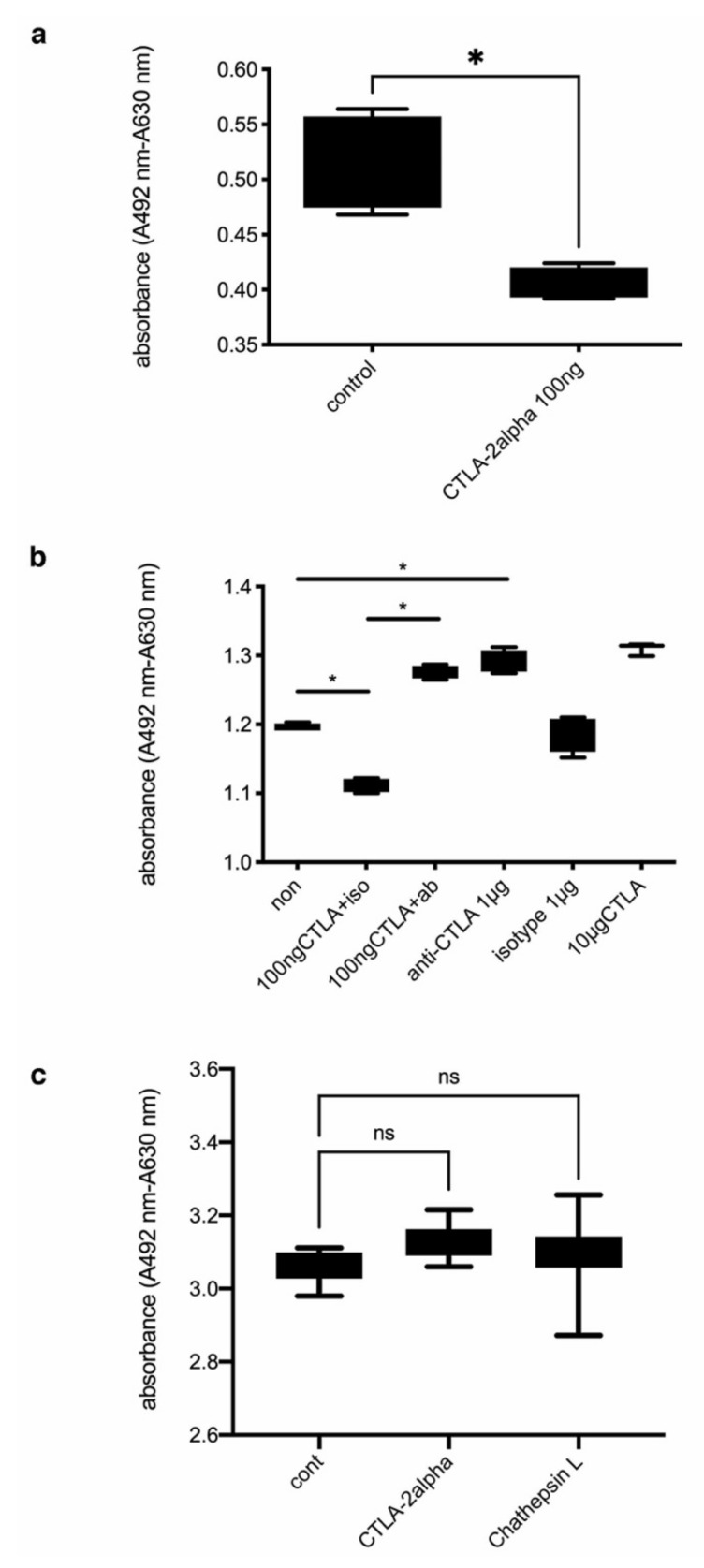
Quantification of mVEC proliferation after treatment with CTLA-2 alpha or anti CTLA-2 alpha neutralizing antibody. (**a**) Proliferation of mVECs following treatment with PBS or CTLA-2 alpha, analyzed using an XTT cell proliferation assay kit. Cell proliferation is indicated by the absorbance (* *p* = 0.0286; *n* = 4). (**b**) Effects of CTLA-2 alpha and an antiCTLA-2 alpha antibody on mVEC proliferation; iso, 1 µg of iso-type control; ab, 1 µg of CTLA-2 alpha neutralizing antibody (* *p* = 0.0286; *n* = 4). (**c**) Effects of CTLA-2 alpha overexpression on mVEC proliferation in vitro (*n* = 5). CTLA-2 alpha, CTLA-2 alpha overexpression in mVECs; Cath L, Cath L overexpression in mVECs; n.s., not significant.

Based on these findings, we posited that CTLA-2 alpha does not regulate the responses of macrophages to inflammation. Therefore, we questioned whether CTLA-2 alpha could directly suppress vascular endothelial cell proliferation. To assess the effects of recombinant CTLA-2 alpha on mVEC proliferation, we used the XTT cell proliferation assay kit. Here, mVECs (4 × 10^3^ cells/mL) were seeded in 96-well flat-bottom cell culture plates containing culture medium with 100 ng/mL CTLA-2 alpha or with PBS. The mVEC proliferation was significantly lower in cultures containing 100 ng/mL CTLA-2 alpha than in those with PBS (*p* = 0.0286; Figure 4a). However, a higher concentration of CTLA-2 alpha did not suppress mVEC proliferation. Therefore, a concentration of 100 ng/mL was used in all subsequent in vitro experiments.

Having found that a low dose of CTLA-2 alpha could suppress mVEC proliferation, we wondered whether the reduction of CTLA-2 alpha by neutralizing antibody would be able to restore mVEC proliferation. We confirmed that 100 ng/mL of CTLA-2 alpha suppressed mVEC proliferation. Interestingly, we found that treatment with anti-CTLA-2 alpha antibody dramatically increased mVEC proliferation compared with the CTLA-2 alpha-treated and PBS-treated groups. In addition, the anti-CTLA-2 alpha antibody accelerated cell proliferation in the same manner as the high concentration (10 µg/mL) of CTLA-2 alpha. These results showed that a low concentration of CTLA-2 alpha has the ability to suppress cell proliferation, and that diminishing the CTLA-2 alpha function improved and accelerated mVEC proliferation (Figure 4b).

In both in vivo and in vitro experiments, we noticed that a high dose of CTLA-2 alpha did not suppress the infiltration of new blood vessels into the cornea or vascular endothelial cell proliferation. To confirm the effects of a high dose of CTLA-2 alpha on mVEC proliferation, mouse CTLA-2-pIRES-EGFP and Cath L expression constructs were generated and introduced individually into mVECs using transfection reagent (Effectene Transfection Reagent; Qiagen). An empty pIRES vector was transfected into mVEC cells as a control. Fluorescence microscopy and quantitative RT-PCR were used to confirm the expression of CTLA-2 and Cath L [30]. Overexpression of CTLA-2 alpha in mVECs did not alter their proliferation compared with either the control or the Cath L overexpression groups. This result demonstrated that a high amount of CTLA-2 alpha had no effect on mVEC proliferation in vitro (Figure 4c).

### 3.5. Detection of CTLA-2 Alpha Expression in the Ocular Tissue

**Figure 5:**
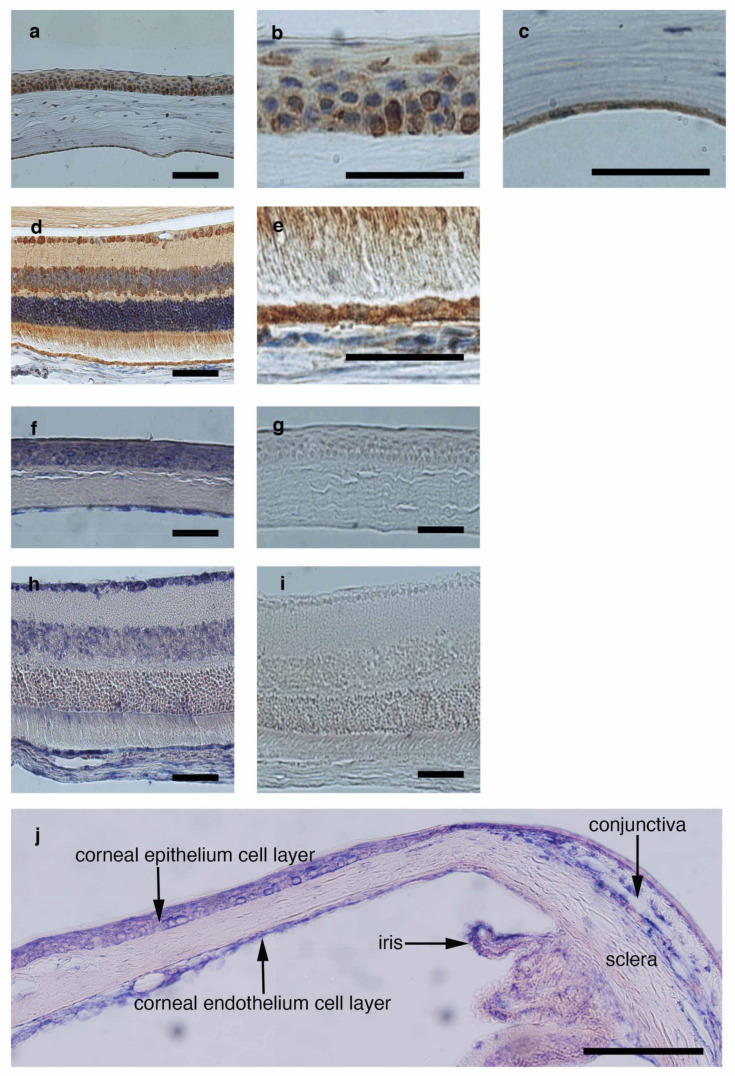
CTLA-2 alpha expression in ocular tissue. CTLA-2 alpha expression was confirmed under histological section assessment with CTLA-2 alpha antibody. Corneal epithelium (**a**,**b**) and endothelium (**a**,**c**) layers highly expressed by CTLA-2 alpha. The whole retinal layer (**d**), especially the retinal pigment epithelial layer (**e**), expressed CTLA-2 alpha. CTLA-2 alpha in situ hybridization in mouse ocular section (**f**–**i**). CTLA-2 alpha specifically expressed in the corneal epithelium (**f**), endothelium (**f**), whole retinal layer (**h**), and RPE layer (**h**). Hybridization with corresponding sense controls did not reveal a signal (**g**,**i**). The location of CTLA-2 alpha in the anterior segment of the eye (**j**). Corneal (cor) CTLA-2 expression was higher than conjunctiva (conj). Scale bar = 200 µm (**a**,**b**,**f**–**i**); 50 µm (**b**,**c**,**e**).

To investigate the expression of CTLA-2 alpha in ocular tissue, we performed both immune histochemistry (Figure 5a–e) and in situ hybridization (Figure 5f–j) analyses to confirm the localization of CTLA-2 alpha in the eye. Intensive staining of CTLA-2 alpha was found at the surface (epithelial cells) and basal layer of the cornea (endothelium cells) and throughout the retina, including the RPE, whereas specific staining was not found for the negative control (sense primer) in in situ hybridization (Figure 5g,i). Moreover, we noticed that CTLA-2 alpha expression in conjunctiva, where a lot of vascular areas exist in the eye, was weaker than corneal tissue in the in situ model (Figure 5j).

## 4. Discussion

We found that CTLA-2 alpha could inhibit angiogenesis in mouse models of CONV and CNV. In addition, CTLA-2 alpha could suppress mVEC proliferation but not the inflammatory response in PECs. A previous investigation showed that CTLA-2 alpha could inhibit Cath L activity, which has been found to promote angiogenesis [19,31]. Specifically, Cath L is highly expressed in endothelial cells and their progenitors, and is essential for matrix degradation and cell invasion during angiogenesis. Here, we provide evidence that CTLA-2 alpha is critically involved in angiogenesis in a model of corneal wounding. Two main findings supported this conclusion: (1) CTLA-2 alpha downregulated mVEC proliferation and (2) blocking CTLA-2 alpha restored mVEC proliferation.

Previous studies have shown that inflammation-induced macrophages play critical roles in inducing angiogenesis in both CONV and CNV [6,32]. Activated macrophages secrete numerous proangiogenic factors such as IL-1beta, TNF-α, and members of the VEGF family [6,28,33,34,35]. Macrophages can differentiate into specific subtypes (e.g., M1 or M2 macrophages) in order to address specific circumstances [36]. M1 macrophages secrete numerous proinflammatory factors such as IL-6, TNF-alpha [28], and IL-1beta [37] in damaged tissues, such as wound areas. However, our results indicated that CTLA-2 alpha did not suppress the inflammatory activation or secretion of proangiogenic factors (e.g., TNF-alpha) in our in vitro experiment. It may be that CTLA-2 alpha suppresses the tissue degradation that stimulates the migration of inflammatory cells into the tissue wound site. Therefore, vessel growth into the cornea or retina should be suppressed by inhibition of matrix degradation, as in Cath L [19]. The mechanisms by which CTLA-2 alpha inhibits angiogenesis remain unclear, and further investigations are necessary to elucidate them.

Cath L is expressed in mVECs [19]. Moreover, a previous report demonstrated that the Cath L protein is highly and specifically expressed in endothelial progenitor cells (EPCs), but not in human vascular endothelial cells (HUVECs) or CD14-positive monocytes [19]. It has been previously reported that Cath L expressed in EPCs plays a critical role in intraocular angiogenesis [31]. Additionally, a specific Cath L inhibitor suppressed HUVEC proliferation. Thus, even fully differentiated endothelial cells such as HUVECs are affected by Cath L activity. Moreover, Cath L deficiency is associated with hair loss in mice, suggesting that Cath L plays a role in the regulation of cell proliferation and differentiation in the skin [38]. It has been reported that CTLA-2 alpha is a Cath L inhibitor [15]. The current findings indicated that CTLA-2 alpha suppressed mVEC proliferation via suppression of Cath L activity. Similarly, napsul-Ile-Trp-CHO: 1-Napthalenesulfonyl-Ile-Trp-Aldehyde (NSITC), a specific Cath L inhibitor, acts as a potent suppressor of tumor angiogenesis, both in vitro and in vivo [39]. This novel function was mediated, at least in part, by the inhibition of proliferation and survival of endothelial cells. In our in vitro experiments, the proliferation of mVECs depended on the concentration of CTLA-2 alpha. Specifically, a high concentration of CTLA-2 alpha did not suppress cell proliferation. Further investigation is needed to fully explain these results.

CTLA-2 alpha expression in the eye was specifically localized to non-vascular areas, where it was highly abundant (Figure 5). For example, in the anterior segment of the eye, the cornea had high CTLA-2 alpha expression. In contrast, the conjunctiva, a highly vascularized area, had lower CTLA-2 alpha expression than the cornea. Detailed analysis of CTLA-2a alpha expression at the ocular surface revealed that the CTLA-2 alpha expression pattern changed at the edge of the cornea, especially in the limbal area. This expression pattern resembled that of another antiangiogenic factor, thrombospondin-1 [2], which also induces regulatory immune cells [40]. Therefore, CTLA-2 should play a critical role in preventing pathological angiogenesis in both the cornea and retinal layer. This novel antiangiogenesis factor may be appropriate for several experiments, such as in a tumor progression model. However, the optimal concentration of CLTA-2 alpha for suppressing angiogenesis is not known. Further investigation is needed to develop CTLA-2 alpha as an antiangiogenesis drug.

## 5. Conclusions

Although the details of the molecular mechanism(s) behind the action of CTLA-2 alpha remain to be fully elucidated, our findings indicate that CTLA-2 alpha affects more than one pathway implicated in angiogenesis, and may have dramatically suppressed angiogenesis in the mouse models of both CONV and CNV precisely because of its action on more than one angiogenesis pathway. The results of the current study indicate that CTLA-2 alpha is a promising candidate drug for the treatment of aggressive angiogenesis in ocular tissue, and that CTLA-2 alpha treatments may help prevent loss of vision.

## Data Availability

Maruyama had full access to all of the data in the study and took responsibility for the integrity of the data and the accuracy of the data analysis. Please contact kazuichi.maruyama@gmail.com with your email address.
